# Green Solvent to Substitute Hexane for Bioactive Lipids Extraction from Black Cumin and Basil Seeds

**DOI:** 10.3390/foods10071493

**Published:** 2021-06-28

**Authors:** Soumaya Bourgou, Iness Bettaieb Rebey, Sofiene Ben Kaab, Majdi Hammami, Sarra Dakhlaoui, Selmi Sawsen, Kamel Msaada, Hiroko Isoda, Riadh Ksouri, Marie-Laure Fauconnier

**Affiliations:** 1 Laboratory of Medicinal and Aromatics Plant, Biotechnology Center of Borj-Cedria, BP 901, 2050 Hammam-Lif, Tunisia; rosainess@yahoo.fr (I.B.R.); sofiene.benkaab@uliege.be (S.B.K.); hammamimajdi@hotmail.com (M.H.); saradakhlaoui@gmail.com (S.D.); selmisawsencbbc@gmail.com (S.S.); msaada_kamel@hotmail.com (K.M.); ksouririadh@gmail.com (R.K.); 2 Laboratory of Chemistry of Natural Molecules, Gembloux Agro-Bio Tech, Université de Liège, Passage des Déportés 2, 5030 Gembloux, Belgium; marie-laure.fauconnier@uliege.be; 3 Integrated and Urban Plant Pathology Laboratory, Gembloux Agro-Bio Tech, University of Liège 2, 8 Passage des Déportés 2, 5030 Gembloux, Belgium; 4 Faculty of Life and Environmental Sciences, School of Integrative and Global Majors (SIGMA), and Alliance for Research on Mediterranean and North Africa (ARENA), University of Tsukuba, Tsukuba 3058572, Japan; isoda.hiroko.ga@u.tsukuba.ac.jp

**Keywords:** *Nigella sativa*, *Ocimum basilicum*, PUFA, tocopherols, phenolics, green extraction, bio-based solvent, bioactivities

## Abstract

A comparative study of bioactive lipids extraction from black cumin (*Nigella sativa* L.) and basil (*Ocimum basilicum* L.) seeds using conventional petroleum-based solvent and green solvent 2-methyltetrahydrofuran (MeTHF) was performed. MeTHF extraction allowed obtaining the highest oil yield in black cumin (34%). Regarding fatty acids composition, linoleic acid (61%) and α-linolenic (78%) were relevant in black cumin and basil green and conventionally extracted oils, respectively. Besides, MeTHF allowed obtaining higher tocopherols and total phenolics contents in black cumin (400 mg/kg of oil and 12 mg EGA/g oil) and basil (317 mg/kg oil and 5 mg EGA/g oil) compared to hexane-extracted ones. The content of major phenolic compounds in the two seed oils, *trans*-hydroxycinnamic acid, rosmarinic acid, and thymol was enhanced by MeTHF extraction. Furthermore, MeTHF-extracted oils possess stronger antioxidant activities (radical scavenging, total antioxidant, and β-carotene bleaching activities) and high and similar anti-inflammatory capacity to hexane-extracted oils. In conclusion, the results revealed that MeTHF is efficient to replace hazardous solvents to extract oil from black cumin and basil seeds rich in compounds relevant to the human diet, including essential polyunsaturated fatty acids (n-6 and n-3), tocopherols, and phenolic compounds with improved biological activities.

## 1. Introduction

In recent years, the demand for edible oils is steadily growing due to their importance for the nutritional security of the global population. Functional lipids are responsible for the health benefits of oils. Polyunsaturated fatty acids (PUFA) belong to two main classes, omega-3 and omega-6 which are essential bioactive compounds with special dietary and functional properties. PUFA are precursors that are metabolized to varied lipid mediators such as eicosanoids and docosanoids with known biological activity. Omega-3 fatty acids have anti-inflammatory activity and are associated with the reduction of the risk for chronic diseases [1]. Moreover, vegetable oils include minor components such as tocopherols, phytosterols, and phenolics that are considered value-added products [2]. Phenolic compounds are among the health-promoting phytochemicals. They draw attention due to their antioxidant activity. Dietary intake of these compounds contributes to a reduction in the risk of chronic disorders like cancer and heart diseases. Phenolic compounds including caffeic, vanillic, and chlorogenic acids as well as tyrosol, quercetin, kaempferol, and catechin have been identified in various seed oils [3]. Tocopherols are natural lipophilic with health benefits. They act as potent antioxidants preventing autoxidation in fats. Tocopherols exhibit biological activities such as neuroprotective, anti-cancer, anti-inflammatory, and cholesterol-lowering properties [4].

The most commonly applied methods for the recovery of oil from seeds are conventional methods that involve the use of high volumes of organic and toxic solvents. Hexane and chloroform/methanol mixture are the most employed ones. Due to their high volatility, organic solvents pose a serious problem for human and environmental health [5]. Hexane has been widely used for oil extraction owing to easy oil recovery, narrow boiling point (63–69 °C), and high solubilizing aptitude [6]. However, it is released into the environment and reacts with the pollutants to form ozone and photo chemicals. Likewise, the highly lipophilic nature of hexane makes it soluble in neural lipids. Thus, it became toxic for neural system when inhaled by humans [5]. Consequently, conventional hexane-extraction consumes considerable energy and is environmentally unfriendly. Health perspective, safety, and environmental concerns have started to look for an urgent replacement of this solvent by a more ecologically suitable option without compromising the yield of oil. In recent decades, a new class of solvents has emerged as an alternative and is called “green solvents” [7,8] that are less harmful to human health and the environment without compromising lipid quality. Recently, laboratory tests have shown that different green solvents have the potential to substitute chloroform and hexane in the recovery of lipids, mainly in the extraction of oilseeds and oil with high biological value, such as tocopherols and sterols [9,10,11]. Indeed, *2*-methyltetrahydrofuran (MeTHF) is one of the green solvents with a high potential to replace n-hexane. This bio-based solvent is cyclic ether, generated from carbohydrates derived from lignocellulosic biomass, which represents the most abundant biomass resources on earth [12]. During the process, MeTHF could be recovered and reused allowing cost optimization on solvent losses, as well as on waste disposal. Thus, this solvent could be an interesting option in the industry for the companies who want to get rid of petrochemicals residues in their product or claim a clean label production with low cost. Even if the solvent is not recycled, its disposal would be cheaper than the disposable of hazardous petro-based solvents, such as hexane [12].

Recently, good results in terms of lipids yield extracted using MeTHF were reported [10,11]. Publications in the literature report oil extraction from basil and black cumin seeds by petroleum solvents [13,14,15,16,17]. To the best of our acknowledgment, oil extraction from these two seeds using alternative green solvents has not been undertaken. Thus, in this study green extraction was applied for the recovery of bioactive lipids from basil and black cumin seeds in which MeTHF was chosen as a good suitable green solvent owing to its safe toxicological profile, low environmental impact, and interesting technical properties.

n-Hexane was applied to obtain oils that were used for comparison. The soxhlet extraction, which is recognized as standard analytical practice for the extraction of oils from solid samples was used. The oil yield, fatty acid composition, bioactive compounds profiling, including tocopherols and phenolics, antioxidant and anti-inflammatory activities were analyzed.

## 2. Material and Methods

### 2.1. Plant Material

Black cumin (*Nigella sativa* L.) seeds were obtained from Menzel Temim (Northeast of Tunisia) in July 2018, while basil (*Ocimum basilicum* L.) seeds were purchased from Kairouan (Center of Tunisia) in September 2018. Seeds ground powder was used for oil extraction.

### 2.2. Seed Oil Extraction

Plant material was placed in a cellulose thimble and inserted in the extraction chamber of a Soxhlet apparatus and placed on a distillation flask filled with 250 mL of solvent: n-hexane or MeTHF. Samples were extracted under reflux for 6 h for basil and 8 h for black cumin. After that, the solvents were removed using a rotary evaporator to obtain oils.

### 2.3. Colour

The color parameters (L*, a*, and b* values) of oils were analyzed by means of a colorimeter (Lovibond, PFX-i series). The oil sample was placed in a glass cell against the light source to get color values. The L* represents the lightness (0–100 represents darkness to lightness), a* value represents red/green coordinate (+ to −), and b* value indicates yellowness to blueness (+ to −).

### 2.4. Fatty Acids Analysis

Fatty acids composition investigation was carried out by means of gas chromatography–flame ionization detection (GC-FID) after derivatization to fatty acid methyl esters (FAMEs). The FAMEs analysis was carried out by GC using HP 6890 gas chromatograph (Agilent, Palo Alto, CA, USA) equipped with RT-2560 capillary column (100 m length, 0.25 mm i.d., 0.20 μm film thickness). The carrier gas was Nitrogen. The initial oven temperature was held at 170 °C for 2 min, increased at a rate of 3 °C/min ramp to 240 °C, and finally held there for 15 min. The injector and detector temperatures were 225 °C. Fatty acids identification of black cumin and basil oils was achieved by comparing their retention times with those of certified FAME mix. The results were expressed as the percentage of each fatty acid in the total.

### 2.5. Tocopherols Content

Tocopherols content was determined according to Tasioula-Margari and Okogeri [18] procedure. Initially, samples of 1 g black cumin and basil oils were extracted with two 2.5 mL of absolute methanol. The residue was extracted once more with two 2.5 mL portions of methanol/ isopropanol (80:20, *v/v*). The extracts were combined and then evaporated to dryness. The residue was dissolved in a methanol/isopropanol/hexane mixture (1:3:1, *v/v/v*) and analyzed for tocopherol content using An Agilent Technologies 1100 series liquid chromatography (RP–HPLC) coupled with a UV-Vis multiwavelength detector. Column 250 × 4.6-mm, 5-μm Eclipse XDB-C18 was used for tocopherols separation. The samples were eluted using solvents combination where solvent A was (2% acetic acid in water), B (methanol), C (acetonitrile), and D (isopropanol). The following parameters were used: 95% A/5% B/2 min; 60% A/10% B/30% C/8 min; 25% B/75% C/22 min, and this percentage was held 10 min; 40% C/60% D/10 min; and this proportion was maintained 15 min; 25% B/75% C/2 min, and lastly, 95% A/5% B/3 min. Flow rate = 1 mL/min. The sample injection volume = 20 µL. Compounds were identified by comparing their retention time with those of standards.

### 2.6. Total Polyphenols Content

The polyphenols content of oil samples was assessed using Folin–Ciocalteu reagent method. Firstly, methanol was used to extract total polyphenols from oil samples [19]. Oil (0.5 g) was extracted with 2.5 mL of methanol. Then, the sample was mixed and centrifuged at 14,000 g. The supernatant was collected, and the process repeated two more times. The polyphenols extracts were collected. Then, 125 μL of the sample was dissolved in 500 μL of distilled water and 125 μL of Folin–Ciocalteu reagent and mixed vigorously. Subsequently, 1.25 mL of 7% sodium carbonate was supplemented and distilled water was added. The solution was incubated for 90 min in the dark. Afterward, the absorbance at 760 nm was determined by a UV-vis spectrophotometer. The total polyphenols content was expressed as milligram of gallic acid equivalent per gram (mg GAE/g) of oil.

### 2.7. Identification of Phenolic Compounds by Liquid Chromatography with Diode Array Detector (LC-DAD)

Phenolic compounds were identified using a high-performance liquid chromatography system (consisting of a vacuum degasser, an autosampler, and a binary pump with a maximum pressure of 400 bar; Agilent 1260, Agilent Technologies, Germany). Zorbax Eclipse XDB C18 column (4.6 × 100 mm and 3.5 μm particle size) was used. The temperature of the column = 25 °C. The scanning range for the diode array detector was set at 200–400 nm. Mobile phase flow rate = 0.4 mL/min. A combination of solvent A (methanol) and solvent B (milli-Q water with 0.1% formic acid) was used as the mobile phase. The following gradient elution was used: 0–5 min, 10–20% A; 5–10 min, 20–30% A; 10–15 min, 30–50% A; 15–20 min, 50–70% A; 20–25 min, 70–90% A; 25–30 min, 90–50% A; 30–35 min, return to initial conditions. Sample injection volume = 2 μL. Compounds in oils were identified in accordance with their retention time with those of standards at 254 nm. The quantity of each compound was determined as microgram per gram of oil (μg/g oil).

### 2.8. Biological Activities

#### 2.8.1. Antioxidant Capacity Evaluation

Three different antioxidant models (DPPH assay, Total antioxidant capacity, and β-carotene bleaching test) were employed to evaluate the antioxidant activity of methanol extracts obtained from black cumin and basil oils.

DPPH assay. The potential of methanolic extracts of oils to reduce the free DPPH radical (1,1-diphenyl-2-picrylhydrazyl) was expressed as IC_50_ (μg/mL), the antiradical dose required to cause a 50% inhibition. For that, each methanolic extract of seed oils, was mixed with a methanolic solution of DPPH [19]. The mixture was vigorously mixed and left in darkness for 90 min. The absorbance was measured at 517 nm against pure methanol using a UV/Vis spectrophotometer.

Total antioxidant capacity. The total antioxidant capacity of methanolic extracts was evaluated through the assay of the green phosphate/Mo5+complex [20]. The absorbance was measured at 695 nm against a blank. The total antioxidant activity was expressed as mg GAE/g oil.

β-carotene bleaching test. The antioxidant activity of methanolic extracts of seed oils was determined using the β-carotene/linoleic acid bleaching test [21]. An aliquot of a β-carotene/linoleic acid emulsion was distributed in 96-well plates, and methanolic solutions of the test samples were added. The plates were incubated at 50 °C for 120 min. The absorbance was measured at 470 nm on a multidetection microplate reader. Readings of all samples were performed immediately (t = 0 min) and after 120 min of incubation. The results are expressed as IC_50_ values (µg/mL).

#### 2.8.2. Anti-Inflammatory Activity Evaluation

Firstly, the cytotoxicity of oil samples on macrophage-like cell line RAW 264. 7 was assessed using the resazurin reduction test as described by O’Brien et al. [22]. Fluorescence was measured using an automated 96-well Fluoroskan Ascent FlTM plate reader (Thermo-Labsystems, Thermo Fisher Scientific, Vantaa, Finland) at an excitation wavelength of 530 nm and an emission wavelength of 590 nm.

Then, the anti-inflammatory potential of oil samples was evaluated by producing nitric oxide formed in RAW 264.7 cells. Oils were dissolved in DMSO to obtain stock solutions, which were further diluted to obtain final concentrations where solvent in the culture medium was maintained at 0.1% (*v/v*) to avoid solvent toxicity. For the determination of nitric oxide, RAW 264.7 cells were seeded in 24-well plates at a density of 2 × 10^5^ cells/ well and were allowed to attach for 24 h/ 37 °C. Cells were then treated with various concentrations of oils and stimulated with 100 μg/mL lipopolysaccharide (LPS). Nitrite production in supernatants was measured using the Griess method. A reference curve of nitrite was prepared on a 96-well plate. The nitric oxide produced was determined by measuring the absorbance at 540 nm (Varioskan Ascent plate reader), and by comparison with the standard calibration curve.

### 2.9. Statistical Analysis

All the experiments were repeated in triplicate and the data were expressed as means ± standard deviations. 1-way ANOVA, Post-hoc homogeneous groups Tukey HSD test (*p* < 0.05) was performed to determine significant differences among means of three independent experiments.

## 3. Results and Discussion

### 3.1. Oil Extraction Yield

**Figure 1:**
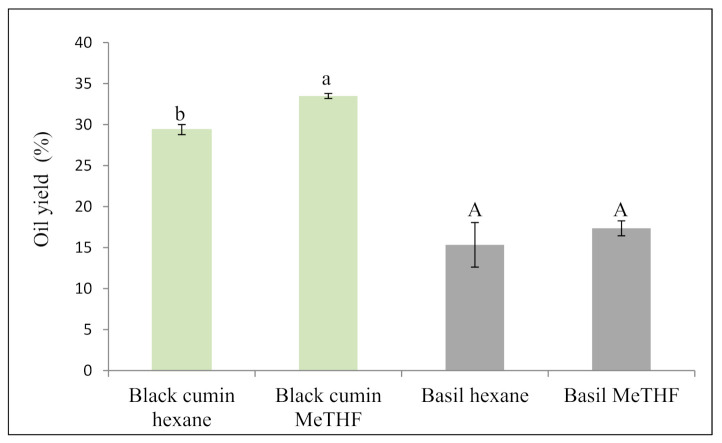
Oil yield of black cumin and basil seeds obtained with conventional and green extractions. Means ± SD, *n* = 3. The means with small letters (a–b) indicates significant differences (*p* < 0.05) between solvents in black cumin and capital letters (A) indicates significant differences (*p* < 0.05) between solvents in basil.

Black cumin and basil seed oils were obtained using new technology including a green solvent MeTHF and compared to conventional hexane extraction. According to Figure 1, black cumin and basil seeds displayed 29 and 15% oil yields, respectively, based on the conventional method. These results are in close agreement with the oil yield of Tunisian black cumin reported by Cheikh-Rouhou et al. [14] after soxhlet extraction using hexane (yield of 28.5%). Moreover, Hamrouni-Sallemi et al. [15] obtained similar results where total lipids content in black cumin seeds was 32% after hexane extraction. On the other hand, basil oil yield found in our study was inferior to obtained by Angers et al. [13], who reported oil yields varying from 20 to 26% according to basil chemotype.

Interestingly, black cumin extraction by bio-based solvent MeTHF recovered an elevated yield (34%) which is better than obtained by hexane (Figure 1). However, there were no significant differences between MeTHF (17%) and hexane for oil extraction from basil seeds. Our results indicate that MeTHF represents a good alternative solvent for lipids extraction from black cumin and basil seeds. These results are in close agreement with the observations after using green solvents extractions, where multiple studies concluded that green solvent had a positive effect on oil yield. Sicaire and al. [8] compared the efficiency of bio-based solvents (MeTHF and limonene) to substitute hexane to extract oil from rapeseeds and reported that MeTHF exhibits a high ability to extract oil and gives comparable yield (47%) to the one obtained by hexane. In some recent works, MeTHF was found more efficient than classic solvent hexane to extract oils. In fact, higher yields were recorded by MeTHF compared to hexane to extract oils from Apiaceae seeds including carvi, anis, and fennel [10,11]. Moreover, Mahmood et al. [23] reported that bio-based solvents ethyl lactate and 2-methyltetrahydrofuran outperformed hexane in terms of microalgal crude lipid yield extraction capacity. MeTHF is an aprotic dipolar solvent with an efficient tool to extract vegetable oils due to the favorable solvent extraction kinetics and successful diffusivity, enabling solvation of a superior quantity of solute at the surface with a more rapid extraction rate than hexane [24]. In this line, De Jesus et al. [9] tested a variety of green solvents to extract oil from wet microalgae *Chlorella pyrenoidosa* and found that MeTHF by soxhlet was more efficient than hexane. The enhancement of oil yield in black cumin using MeTHF in our study could be due to the variable polarity of this solvent, leading to the extraction of different types of lipids at high temperature, including a high concentration of nonpolar or neutral lipids in addition to polar lipids. According to the study of Hamrouni et al. [15], Tunisian black cumin seeds oil is rich in polar lipids (6.5% of total lipids) mainly phospholipids. Thus, we can hypothesize that in our study green solvent enhanced the extraction of polar lipids fraction in black cumin oil leading to higher oil yield.

Overall, our results demonstrate that MeTHF may become a suitable substitute for hexane in terms of oil extraction yield. Additionally, MeTHF is derived from a renewable source and is accepted as safe. Its use in pharmaceutical chemical processes has been agreed [25].

### 3.2. Colour Profile

The CIELAB color parameters of the oils (L*, a*, and b*) from black cumin and basil seeds are shown in Table 1. The color parameters were reported as L* (lightness to darkness), a* (greenness to redness), and b* (yellowness to blueness).

The conventional extracted from black cumin and basil oils showed high L* and b* values. Our results are in accordance with those of Cheikh-Rouhou et al. [14] and Ghaleshahi et al. [26] who showed that black cumin oil presented a high b* value. Green extracted black cumin and basil oils presented lower L* but higher a* values. Moreover, b* increased slightly in basil oil compared to hexane extracted. The enhancement in a* value (indicates the brilliant red color of oil) may be due to the improvement in the level of carotenoids pigments that give red color in oil. In previous reports, MeTHF showed a higher extraction yield for carotenoids than n-hexane due to its property to dissolve carotenoids [24].

### 3.3. Fatty Acid Composition

The fatty acid profile of the oil seeds obtained with conventional and green extraction from black cumin and basil is summarized in Table 2. The results revealed that the two oils were especially rich in PUFA with levels of 61 and 78% in black cumin and basil, respectively. Moreover, black cumin oils were characterized by high monounsaturated fatty acids (MUFA) amounts with a percentage of 19% obtained with conventional and green extractions.

As shown in Table 2, a comparison of fatty acids composition revealed differences between hexane and green-extracted oils. Linoleic acid (C18:2) is the marker fatty acid of black cumin seeds hexane and MeTHF oils, while α-linolenic acid followed by linoleic acid were the major ones in basil oils. Moreover, oleic and palmitic acids were present at high levels in black cumin and basil oils at similar levels in green and hexane-extracted oils. However, MeTHF improved the extraction of behenic acid in black seed oil while the percentages of stearic, arachidic, and eicosenoic acids were reduced. The similarity in the fatty acid composition of hexane-extracted and green-extracted oils by MeTHF had been reported in *Carum carvi*, anise, fennel, and rapeseed seeds [8,10,11].

On the other hand, our results are similar to those found by Cheikh-Rouhou et al. [14] and Sellami Hamrouni et al. [15] indicating that black cumin oil is dominated by linoleic acid followed by oleic acid, whereas linolenic (C18:3) and arachidic (C20:0) acids were not identified in black cumin seeds from Germany [27]. Besides, basil seeds have been reported to hold an elevated level of α-linolenic acid (ALA, C18:3) ranging from 43 to 75% according to the origin [13,17]. Essential fatty acids are obligatory for health. They cannot be produced in the human body and must be provided through the diet. Linoleic and linoleic acids are essential fatty acids that can reduce the incidence of coronary heart diseases and lower high blood pressure. Moreover, they have been shown to improve skin barrier function and dermatitis [28].

On the other hand, a low PUFA/SFA ratio in food (below 0.45) is a risk factor for augmented blood cholesterol levels. In this study, PUFA/SFA ratios in green extracted oils were high; about 3 and 6 for black cumin and basil oils, respectively. Moreover, these ratios are similar to those obtained in oils extracted conventionally (Table 2). Thus, these results indicated that the green extracted black cumin and basil oils have appropriate and balanced fatty acid compositions with suitable connotations to the human diet. Besides, oils with elevated content of PUFA, especially linoleic and linolenic acids, are a target for skincare products in the cosmetic and pharmaceutical sectors [27].

### 3.4. Tocopherols Contents

Apart from triacylglycerols as major components in black cumin and basil oils, there are also some minor components like tocopherols and polyphenols that could be recognized as micronutrients. Consumption of different isomeric forms of tocopherols like α-, β-, γ- and δ- contribute substantially towards the health improvement. Tocopherols have main immunomodulatory functions and can delay the pathogenesis of a variety of degenerative illnesses. They are effective as lipid antioxidants. The activity is linked mostly to their capacity to scavenge peroxyl radicals and so break free chain reactions [27].

Analysis of the tocopherols in seed oils extracted using conventional solvent hexane (Table 3) showed that the γ-isomer (135 mg/kg) followed by α-isomer (114 mg/kg) was the principal tocopherols in black cumin seed oil while basil seeds oil contained high amounts of γ-tocopherol (238 mg/kg). Small amounts of α and β- tocopherols were recorded in the last oil, however, δ-isomer was not detected in basil oil. Most seed oils contained α-tocopherol, β-tocopherol, and γ-tocopherol [29]. Hassanien et al. [30] reported also that γ-tocopherol (939 mg/g) followed by α-isomer, were the most important tocopherols in the oil of black cumin seed from Egypt. According to Ramadan et al. [27], α-tocopherol (284 µg/mg) and γ-tocopherols (225 µg/g) were the major components in black cumin seed oil. Regarding *O. basilicum*, γ-tocopherol was identified as being the one quantified at high concentration in seed oil [2,26]. Moreover, it was reported that γ-tocopherol is a characteristic compound in seed oils of species belonging to the *Lamiaceae* family [31].

As can be seen in Table 3, MeTHF was found to favor the extraction of tocopherols from black cumin seeds while tocopherol content in basil oil resulted from MeTHF extraction was comparable to the one gets by conventional solvent employing hexane. Indeed, an increase of the amounts of major isomers α and γ by 15 and 22% were recorded in green extracted black cumin oil compared to the hexane-extracted oil (Table 3). Moreover, an enhancement of the amounts of quantities of minor isomers β, and δ–tocopherols by 0.5 and 2 folds were found also (Table 3). Extraction of minor compounds including tocopherols by MeTHF as green solvent has been studied. Sicaire et al. [8] described that total tocopherols content in rapeseed oil after extraction by means of MeTHF and n-hexane were close. Recently, MeTHF was an excellent alternative to hexane to extract oil from *Opuntia ficus–Indica* L. seeds with similar tocopherols contents [32].

In brief, MeTHF proposed in this study, in addition to constitutes an ecological friendly alternative, permit obtaining the greatest tocopherols content in black cumin and basil seed oils. The study proved that the use of green solvents guarantees little chemical toxicity and elevated bioactive compounds extraction effectiveness.

### 3.5. Total Phenolic Content

Phenolic compounds have a great effect on the sensory and nutritional quality of oils and may protect against lipid oxidation through quenching of radical reactions. Oils containing phenols may have the potential for health-improving particularly in the prevention of heart illnesses [33].

The total phenolic content (TPC) of the two seed oils was analyzed. As given in Table 4, conventional oils extracted by hexane displayed interesting phenolic amounts (10 and 4 mg GAE/g oil in black cumin and basil oils, respectively). Lower contents of TPC were reported by Ramadan [34] and Ghaleshahi et al. [26] in cold-pressed oil from seeds of black cumin from Egypt and oil from Iranian basil seeds, respectively. The amount of total polyphenols content is recognized to depend on the polarity of the extraction solvent and on additional factors, such as plant cultivar, degree of seed maturation, environmental fluctuations, and growing site [33].

As reported in Table 4, a comparison of conventional and green extraction indicated that black cumin and basil seed oils obtained using green solvent MeTHF showed higher TPC (12 and 5 mg GAE/g oil, respectively) than those extracted by organic solvent hexane. Thus, the bio-based solvent proposed in this work allowed obtaining the maximum polyphenols content in black cumin and basil seed oils. These data imply that green extracted oils include the main level of phenolic components which may actively participate in the oil stability under accelerated oxidative stress. Recently, MeTHF proved to be an effective alternative to petroleum-based solvent to extract oil-enriched phenolic compounds. Indeed, Bourgou et al. [11] reported that the employ of MeTHF ameliorates the extraction of total phenolics in carvi seeds oil by 1.6 folds when compared to hexane. Besides, anise and fennel seed oils extracted by means of MeTHF exhibited drastically advanced TPC than those extracted with conventional solvents including chloroform/methanol and hexane [10]. According to Ravi et al. [6], black soldier fly oil extracted by MeTHF had polyphenols amount that was 2.5 times superior to that extracted by n-hexane.

### 3.6. Phenolic Compounds Identification

It is interesting to identify antioxidant compounds in the black cumin and basil seeds oils since they may contribute to the quality and health benefits of the oils. Analysis of phenolic compounds in seed oils extracted using conventional and green solvents allowed the identification of 15 phenolic compounds. The compounds include 9 phenolic acids, 4 flavonoids, and two oxygenated monoterpenes (Table 5).

Black cumin oil extracted using hexane was characterized by a high amount of sinapic acid (16 µg/g oil). In addition, *trans*-hydroxycinnamic acid (5 µ/g oil), quercetin (6 µg/g oil), and isorhamnetin (7 µg/g) were also identified with important levels. Thymoquinone, a typical monoterpene of black cumin was identified at a low concentration (0.1 µg/mL). This monoterpene ketone was already identified in Tunisian black cumin essential oil [35]. However, thymoquinone level was lower than that recorded in Turkish black cumin cold-pressed and soxhlet extracted oils (6 and 14 µg/g oil, respectively [16]. In addition, these authors identified benzoic acid as the most relevant phenolic compound in the oil with content varying from 2 to 4 µg/g oil according to extraction procedure. In the case of basil oil, a high amount of phenolic monoterpene thymol was detected in the oil (33 µg/g oil). Moreover, interesting amounts of luteolin (9 µg/g oil) and ellagic acid (4 µg/g oil) were recorded. In line with our results, thymol has also been cited recently as the key polyphenolic compound in basil seeds oil (11 mg/100g oil) obtained after extraction through a combination of n-hexane and ethyl acetate [2].

Green extracted oils showed a clear difference compared to conventional extracted ones (Table 5). Interestingly, MeTHF extracted black cumin and basil oils have richer phenolic profiles than hexane-extracted ones. In fact, new compounds were identified in consequence of biobased extraction in the two seeds oils including high amounts of gallic acid (5 and 7 µg/g oil) and chlorogenic acid (4 and 5 µg/g oil), as well as small levels of syringic acid (1 µg/g oil) in black cumin and basil oils, respectively. In addition, MeTHF was efficient to extract ferulic acid in basil oil (1 µg/g oil).

Except for sinapic acid which was not detected in black cumin green extracted oil, the results indicate that MeTHF provides oils with similar or higher contents of individual compounds compared to conventional extracted oils. Indeed, MeTHF was found to favor the extraction of *trans*-hydroxycinnamic acid and thymoquinone in black cumin which contents are enhanced by 6 and 4 times compared to conventionally extracted oil. In the same line, thymol, rosmarinic acid, and circimaritin amounts increased by about 4, 105, and 6 times in green extracted basil oil compared to hexane-extracted oil. These results may be related to the relative solubility of phenolic metabolites in both solvents. In accordance with our results, Nutrizio et al. [36] assessed recently the solubility of wild thyme bioactive compounds in different green solvents and showed that MeTHF exhibit a higher capacity to dissolve thymol compared to hexane. In another study, MeTHF was reported to recover terpenes such as limonene [37] and carvone [38] from plant matrix as efficiently as hexane with an increased yield. Recently, Combes et al. [39] tested aprotic and polar bio-compatibles solvents to extract molecules such as *p*-hydroxycinnamic acid and demonstrated that 2-MeTHF can be an excellent candidate to extract *p*-coumaric acid. Besides, Wang et al. [40] explored a green and low-cost alternative method for the extraction and separation of flavones baicalin and baicalein from *Scutellaria baicalensis Georgi* and reported that MeTHF was found to be efficient to recover flavones from the plant material after ionic liquids extraction with more than 97% baicalein being recovered.

Thus, agro solvent MeTHF demonstrated the potential to be a better alternative to petroleum solvent n-hexane applied in industry, in terms of phenolic profile.

### 3.7. Antioxidant Activity

In this study, the antioxidant activities of black cumin and basil seed oils obtained by conventional and green solvents were investigated. Generally, the two seed oils extracted by the conventional extraction method by means of hexane possess important antioxidant activity (Table 4). Indeed, black cumin seed oil showed the best antiradical activity (IC_50_ = 1.7 mg/mL), while basil oil showed the highest total antioxidant activity (16.6 mg GAE/g of oil). Moreover, the two oils exhibited a significant and similar ability to inhibit the bleaching of β-carotene (IC_50_ about 3 mg/mL).

Interestingly, black cumin and basil oils extracted with MeTHF showed significantly higher antioxidant activity than the ones extracted conventionally (Table 4). In fact, the total antioxidant was enhanced in the two oils seeds by about 4 folds while the bleaching capacity of β-carotene increased by 3 and 1.6 folds in black cumin and basil seeds oils, respectively. In the case of antiradical activity, it increased in the two oils, especially in basil one by 2.8 folds compared to the oil extracted with hexane. In line with Ravi et al. [6], the use of MeTHF improved the antioxidant capacity of black soldier fly oil compared to the one extracted by hexane. Besides, more bioactive compound levels and improved antioxidant activity were recovered in anise and fennel oil seeds extracted by MeTHF solvent compared to conventionally extracted oils [10]. Former studies, conducted by us [11] have revealed that green extracted carvi oil obtained using MeTHF displayed stronger antiradical activity than the oils extracted conventionally.

Likewise, it is clear that the higher antioxidant power of green extracted oils compared to hexane-extracted ones could be explained by the different phenolic compositions. In our study, MeTHF extracted oils were specifically rich in phenolic acids. These compounds have been reported as strong antioxidant agents and effective intracellular ROS scavengers. The effectiveness of phenolic compounds as antioxidants depends on numerous factors, such as the number of hydroxyl groups bonded to the aromatic ring, the site of bonding, and the mutual position of hydroxyls in the aromatic ring [41]. In our study, black cumin oil green extracted oil is rich in hydroxycinnamic acid which was known for its in vitro antioxidant activity [41]. Moreover, green extracted basil oil presented high content of gallic and ellagic acids and especially rosmarinic acid. Phenolic acids with three (gallic acid) or two hydroxyl groups (rosmarinic acid) bonded to aromatic rings in the ortho position showed strong antioxidant and anti-radical activity [41]. Moreover, minor compounds like thymoquinone can play a part in the antioxidant activity of black cumin oils. This ketone was already reported as a strong antioxidant compound exhibiting potent in vitro and ex-vivo antioxidant activities [35]. On the other hand, tocopherols present in the green extracted black cumin and basil oils in our study could act as antioxidants also. Tocopherols are known to contribute to oil’s oxidative stability and are also responsible for their antioxidant activity. The efficiency of tocopherols as lipid antioxidants has been ascribed primarily to their aptitude to break chain reactions by reacting with fatty acid peroxy radicals [27].

Overall, our results suggest that MeTHF could be an excellent substitute for hexane to recover black cumin and basil oils with enhanced antioxidant activity. Beyond the health benefits related to polyphenols intake from foods, the significance of producing oils with prominent phenolic compound contents and antioxidant activity is associated also with the fact that oils rich in PUFA are very vulnerable to oxidative degradation. Thus, the enhanced bioactivity in green extracted black cumin and basil oil might facilitate decreasing lipid peroxidation at ambient and elevated temperatures and could be so used as functional oils for dietary supplements.

### 3.8. Anti-Inflammatory Activity

**Figure 2:**
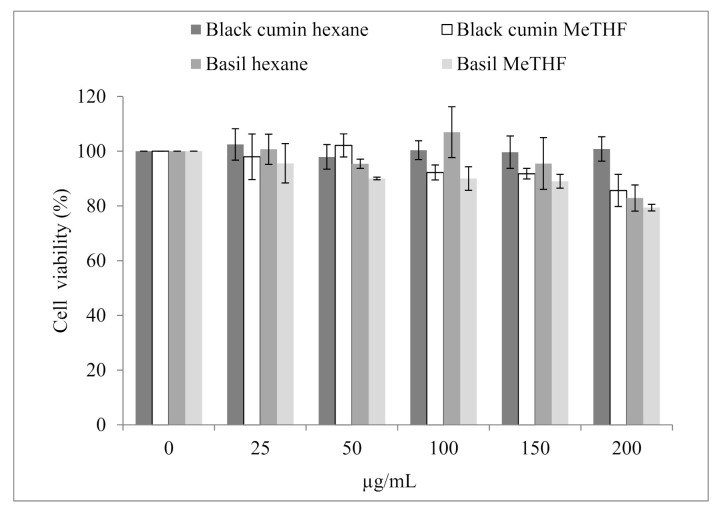
Effect of black cumin and basil oils at different concentrations (25–200 µg/mL) obtained with conventional and green extractions on the viability of RAW 264.7 cells. Means ± SD, *n* = 3.

Firstly, the cytotoxicity effect of conventional and green extracted oils on RAW 264.7 cells was investigated using the resazurin test. After 24 h of incubation with a wide range of concentrations, no significant cytotoxic effects of the four oils to RAW 264.7 cells were detected at up to 150 μg/mL concentrations (Figure 2). At 200 µg/mL, conventional and green extracted oils inhibited cell viability, reaching 21% for basil MeTHF-extracted oil. Based on these data, the subsequent anti-inflammatory activity of these oils at concentrations (25–150 µg/mL) was assessed using LPS stimulated RAW 264.7 macrophages.

A well-known inflammatory marker, specifically NO, was chosen in order to assess the anti-inflammatory activity of black cumin and basil oils. In macrophages and invading immune cells, the elevated quantity of NO generated by inducible nitric oxide synthase (iNOS) following LPS and/or inflammatory cytokines plays a central function in inflammation.

**Figure 3:**
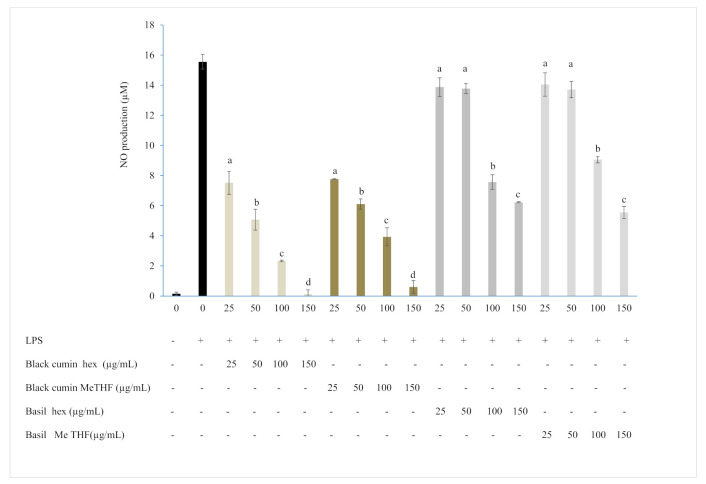
Effect of black cumin and basil oils obtained with conventional and green extractions on NO overproduction in LPS-stimulated RAW264.7 macrophages. The means with small letters (a–d) indicates significant differences between concentrations of solvent in the same species (*p* < 0.05).

Cells challenged with LPS trigger an important NO accumulation (16 μM), however, the incubation of murine macrophages with a graded concentration of hexane and MeTHF-extracted oils induced inhibition of NO production in a concentration-dependent manner as it is depicted in Figure 3. Black cumin exhibited higher activity than basil. At a low concentration of 25 µg/mL, black cumin hexane and MeTHF extracted oils showed potent activity inhibiting NO release by 52 and 50%, respectively, while basil showed low activities at these concentrations which did not exceed 12%. At high concentrations of 100 and 150 µg/mL, NO production inhibition reached 85 and 99% by black cumin oil extracted using hexane and 75 and 96% by black cumin MeTHF-extracted oil. For this species, IC_50_ values were 22 and 25 µg/mL, in hexane and MeTHF-extracted oils, respectively. In the case of basil, the highest activity was achieved at 150 µg/mL, in fact. NO production decreased by 60 and 64% in hexane and green extracted basil oil, respectively.

Independently to the species, the results showed similarity of anti-inflammatory activity in hexane and green extracted oils which suggest that the green solvent is as able as hexane to extract anti-inflammatory compounds from the two plant seeds. Our data are in accordance with previously published work showing that some MeTHF extracted oils exhibited important anti-inflammatory activity [11,42]. The activity could be the results of additive and/or synergistic interaction between compounds or the action of specific compounds. For example, thymoquinone which is well-known to be responsible for the anti-inflammatory power of *Nigella sativa* at low level (inhibited NO production by 95.0% at very low concentration (4.1 μg/mL) [35], was efficiently extracted using hexane and green solvent with higher content in green extracted oil. This ketone produces its suppression effect on NO release via the inhibition of inducible NOS mRNA and protein expressions as well as via its powerful direct antioxidant activity as a ROS scavenger [35]. However, hexane-extracted oil contains a high amount of sinapic acid (absent in green extracted oil) which could participate in the activity of the hexane extracted oil [43].

Furthermore, other minor compounds mainly phenolics could be involved in the bioactivity of black cumin and basil oils. For instance, the abundance of phenolic acids in the seed oils including *trans*-hydroxycinnamic and rosmarinic acids could probably participate to their anti-inflammatory activity since they can inhibit the NO generation and iNOS expression in LPS-stimulated Raw 264.7 [43].

## 4. Conclusions

The results show that the biobased solvent MeTHF is a promising green alternative to hexane to recover enhanced functional oils from black cumin and basil seeds. The black cumin oil yield extracted using bio-based solvent was increased compared to conventional extraction. Green extracted oils were rich in PUFA mainly linoleic (61%) and α-linolenic (56%) acids. For both seeds, the oils extracted with MeTHF gave the highest tocopherols and total polyphenols contents as well as the most pronounced in vitro antioxidant capacity. Green extracted oils possess potent anti-inflammatory activity also. The content of individual phenolic compounds including phenolic acids, flavonoids, and monoterpenes was enhanced after green-extraction. Taken together, our results show that using a substitute solvent to extract oil from black cumin and basil seeds would be beneficial to obtain oils with enhanced bioactive compounds content and functional properties which can lead to additional innovations and their integration in cosmetic and beneficial formulations. Biobased-solvent MeTHF has the advantages to be eco-friendly, safe with biodegradability, and easily recycled power. Being economical, it shows a mainly competitive edge when it comes to the economical equation of extraction procedure compared to other green technologies. Due to the initial high costs of purchasing and setting up the equipment, the efficiency of other technologies such as supercritical carbon dioxide extraction is hindered. Finally, further study must be undertaken using a cellular model to verify the protection ability of green extracted oils to protect cells against oxidative cell damage. Moreover, storage effects on oil quality needs to be conducted to explore oxidative stability of the MeTHF-extracted oils.

## Figures and Tables

**Table 1:** Color parameters (CIELAB) of black cumin and basil seeds oils obtained with conventional and green extractions.

	Black Cumin		Basil	
	Hexane	MeTHF	Hexane	MeTHF
L*	90.6 ± 0.1 ^a^	27.0 ± 0.3 ^b^	59.9 ± 1.0 ^A^	32.8 ± 0.6 ^B^
a*	−0.13 ± 0.0 ^b^	3.9 ± 0.1 ^a^	−0.5 ± 0.1 ^B^	3.4 ± 0.6 ^A^
b*	86.6 ± 1.2 ^a^	5.76 ± 0.5 ^b^	37.0 ± 1.0 ^B^	42.3 ± 0.5 ^A^

means ± SD, *n* = 3. The means with small letters (a–b) indicates significant differences (*p* < 0.05) between solvents in black cumin and capital letters (A–B) indicates significant differences (*p* < 0.05) between solvents in basil.

**Table 2:** Fatty acids composition (%) of black cumin and basil oils obtained with hexane and MeTHF extractions.

Fatty Acids (%)	Black Cumin		Basil	
	Hexane	MeTHF	Hexane	MeTHF
C16:0	12.5 ± 0.3 ^a^	13.7 ± 0.2 ^a^	8.3 ± 0.2 ^A^	8.4 ± 0.1 ^A^
C18:0	3.8 ± 0.9 ^a^	2.0 ± 0.2 ^b^	3.4 ± 1.2 ^A^	3.4 ± 0.4 ^A^
C18:1 n-9	18.9 ± 0.5 ^a^	19.4 ± 0.2 ^a^	8.2 ± 0.3 ^A^	8.6 ± 0.2 ^A^
C18:2 n-6	61.1 ± 0.3 ^a^	60.8 ± 0.1 ^a^	22.8 ± 0.7 ^A^	23.4 ± 0.2 ^A^
C18:3 n-3	0.3 ± 0.1 ^a^	0.3 ± 0.0 ^a^	56.0 ± 2.2 ^A^	54.9 ± 1.2 ^A^
C20:0	0.2 ± 0.0 ^a^	0.1 ± 0.1 ^b^	1.4 ± 1.2 ^A^	1.3 ± 1.1 ^A^
C20:1	0.4 ± 0.1 ^a^	0.1 ± 0.1 ^b^	-	-
C22:0	2.9 ± 0.1 ^b^	3.6 ± 0.1 ^a^	-	-
PUFA	61.4 ± 0.4 ^a^	61.1 ± 0.1 ^a^	78.7 ± 2.2 ^A^	78.3 ± 1.4 ^A^
MUFA	19.3 ± 0.6 ^a^	19.6 ± 0.3 ^a^	8.2 ± 0.3 ^A^	8.6 ± 0.6 ^A^
SFA	19.3 ± 1.3 ^a^	19.3 ± 0.4 ^a^	13.0 ± 2.6 ^A^	13.1 ± 1.6 ^A^
PUFA/SFA	3.2	3.2	6.1	6.1

means ± SD, *n* = 3. The means with small letters (a–b) indicates significant differences (*p* < 0.05) between solvents in black cumin and capital letters (A) indicates significant differences (*p* < 0.05) between solvents in basil. Palmitic acid (C16:0), stearic acid (C18:0), oleic acid (C18:1 n-9), linoleic acid (C18:2 n-6), α-linolenic acid (C18:3 n-3), arachidic acid (C20:0), eicosenoic acid (C20:1), behenic acid (C22:0), MUFA, monounsaturated fatty acids; PUFA, polyunsaturated fatty acids; SFA, saturated fatty acids.

**Table 3:** Composition of tocopherols (mg/kg of oils) in black cumin and basil seed oils after extraction by hexane and MeTHF.

	Black Cumin		Basil	
	Hexane	MeTHF	Hexane	MeTHF
α-Tocopherol	113.8 ± 7.0 ^b^	131.0 ± 7.4 ^a^	33.7 ± 0.3 ^B^	43.7 ± 7.0 ^A^
γ-Tocopherol	134.8 ± 6.3 ^b^	164.2 ± 6.3 ^a^	237.7 ± 3.3 ^A^	231.5 ± 8.4 ^A^
β-Tocopherol	44.1 ± 4.7 ^b^	73.5 ± 7.2 ^a^	20.4 ± 3.2 ^B^	42.2 ± 7.1 ^A^
δ-Tocopherol	15.1 ± 3.2 ^b^	32.6 ± 4.7 ^a^	nd	nd
Total tocopherols	307.7 ± 7.1 ^b^	400.7 ± 6.4 ^a^	292 ± 0.36 ^A^	317 ± 8.1 ^A^

nd: not detected. means ± SD, *n* = 3. The means with small letters (a–b) indicates significant differences (*p* < 0.05) between solvents in black cumin and capital letters (A) indicates significant differences (*p* < 0.05) between solvents in basil.

**Table 4:** Total polyphenols contents and radical scavenging capacity of black cumin and basil oils obtained with hexane and MeTHF extractions.

	Black Cumin		Basil	
	Hexane	MeTHF	Hexane	MeTHF
TPC (mg GAE/g of oil)	9.3 ± 0.3 ^b^	11.6 ± 0.1 ^a^	4.4 ± 0.2 ^B^	5.3 ± 0.1 ^A^
DPPH radical activity (IC_50_ mg/mL)	1.7 ± 0.2 ^a^	1.3 ± 0.3 ^a^	3.4 ± 0.6 ^A^	1.2 ± 0.3 ^B^
Total antioxidant capacity (mg GAE/g of oil)	12.9 ± 1.0 ^b^	64.2 ± 2.1 ^a^	16.6 ± 1.3 ^B^	67.4 ± 2.5 ^A^
β-carotene bleaching activity (IC_50_ mg/mL)	3.9 ± 0.4 ^a^	1.1 ± 0.3 ^b^	3.0 ± 0.1 ^A^	1.8 ± 0.3 ^B^

means ± SD, *n* = 3. The means with small letters (a–b) indicates significant differences (*p* < 0.05) between solvents in black cumin and capital letters (A–B) indicates significant differences (*p* < 0.05) between solvents in basil.

**Table 5:** Phenolic compounds identification (µg/g oil) in black cumin and basil seed oils after extraction by hexane and MeTHF.

	RT (min)	Black Cumin		Basil	
		Hexane	MeTHF	Hexane	MeTHF
Gallic acid	8.2	nd	4.9 ± 0.3	nd	7.3 ± 0.1
Chlorogenic acid	15.7	nd	3.9 ± 0.1	nd	7.2 ± 1.2
Caffeic acid	17.2	nd	nd	0.7 ± 0.1 ^B^	1.6 ± 0.1 ^A^
Syringic acid	17.8	nd	0.9 ± 0.1	nd	0.8 ± 0.1
Sinapic acid	19.6	16.3 ± 0.5	nd	nd	nd
Ferulic acid	20.2	nd	nd	nd	1.4 ± 0.1
*trans*-hydroxycinnamic acid	20.9	5.0 ± 0.1 ^b^	28.6 ± 1.1 ^a^	nd	nd
Rosmarinic acid	21.1	nd	nd	0.2 ± 0.1 ^B^	21.1 ± 0.2 ^A^
Ellagic acid	22.8	nd	nd	3.5 ± 1.5 ^B^	6.3 ± 0.1 ^A^
Luteolin	23.7	nd	nd	9.4 ± 0.1 ^A^	9.4 ± 0.1 ^A^
Quercetin	24.2	5.7 ± 0.3 ^b^	6.7 ± 0.4 ^a^	nd	nd
Thymoquinone	24.8	0.1 ± 0.0 ^b^	0.35 ± 0.1 ^a^	nd	nd
Circimaritin	25.1	nd	nd	1.0 ± 0.1 ^B^	5.8 ± 0.2 ^A^
Isorhamnetin	25.6	6.6 ± 0.1 ^a^	6.3 ± 0.2 ^a^	nd	nd
Thymol	26.1	nd	nd	33.1 ± 0.3 ^B^	127.6 ± 0.8 ^A^

nd: not detected. means ± SD, *n* = 3. The means with small letters (a–b) indicates significant differences (*p* < 0.05) between solvents in black cumin and capital letters (A–B) indicates significant differences (*p* < 0.05) between solvents in basil.
